# Neonatal hypocomplementemic urticarial vasculitis

**DOI:** 10.1093/rap/rkab090

**Published:** 2021-11-24

**Authors:** Carine J Moezinia, James Alden

**Affiliations:** Rheumatology Department, Wexham Park Hospital, Frimley Health NHS Foundation Trust, Slough, UK

A 3-day-old boy presented with a 1 day history of a rapidly progressing palpable purpuric rash on both feet (Fig. 1). Neonatal examination at birth had been unremarkable. The rash was limited to the feet and he was clinically well otherwise, with normal observations. His mother, as well as several paternal cousins, two uncles and an aunt, had a history of similar rashes. These reportedly appear and resolve spontaneously over a period of a few days, are purpuric in nature and episodes recur approximately every 6 months. A complete blood count, liver and renal function tests, CRP and coagulation screen were unremarkable. Both C3 and C4 levels were decreased. A diagnosis of hypocomplementemic urticarial vasculitis (HUV) was made.

**Fig. 1:**
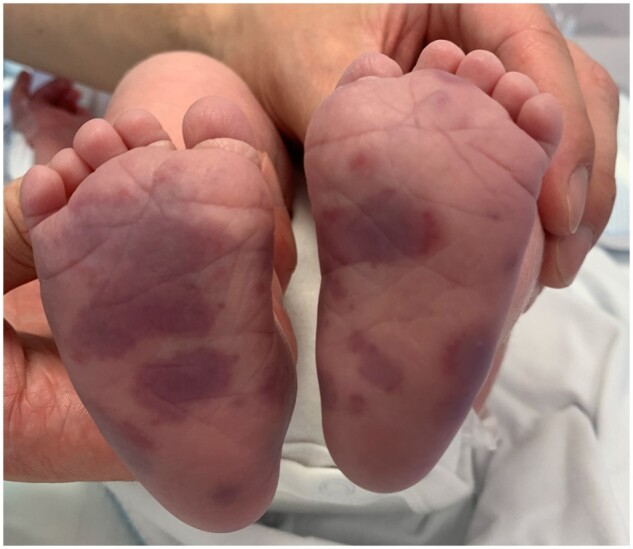
Palpable purpuric rash of hypocomplementemic urticarial vasculitis in a 3-day-old neonate.

HUV syndrome is a syndrome of recurrent urticarial vasculitis, arthralgias/arthritis and hypocomplementemia. It can also involve the kidneys, gastrointestinal tract, eyes, lungs and nervous system. It is a condition well described in adults, but rarely presents in children. HUV can occur with predominantly cutaneous manifestations and few or no systemic features and is associated with C1q antibodies [1]. HUV is generally sporadic, however, familial cases have been described with mutations in DNASE1L3 [2]. The pattern of inheritance for familial cases associated with this mutation is autosomal recessive, which contrasts with this family, who follow an autosomal dominant pattern. No treatment was initiated in this patient and the lesions spontaneously resolved after a few days.

*Funding*: No specific funding was received from any bodies in the public, commercial or not-for-profit sectors to carry out the work described in this article.

*Disclosure statement*: The authors have declared no conflicts of interest. Written informed consent was provided for the publication of this article.

## Data availability statement

Data are available upon reasonable request by any qualified researchers who engage in rigorous, independent scientific research, and will be provided following review and approval of a research proposal and Statistical Analysis Plan (SAP) and execution of a Data Sharing Agreement (DSA). All data relevant to the study are included in the article.
